# Effects of different factors on fly ash-based functional soil and its oat grass cultivation

**DOI:** 10.3389/fpls.2022.1048101

**Published:** 2022-12-02

**Authors:** Tengteng Liu, Fenglan Han, Zhibing Xing, Jiaqi Wang, Xiongwei Dong, Changcong An

**Affiliations:** ^1^ Institute of Materials Science and Engineering, North Minzu University, Yinchuan, China; ^2^ International Scientific & Technological Cooperation Base of Industrial Waste Recycling and Advanced Materials, Yinchuan, China

**Keywords:** Fly ash, functional soil, ecological restoration, Stroma, Mixture ratio, plant

## Abstract

Using fly ash as the main matrix for plant ecological restoration is effective for constructing a sustainable and ecological environment. The relevant properties of functional soil change due to different factors. Based on the orthogonal experiment of functional soil and the pot experiment of oat grass, fly ash was used as the matrix material for functional soil. Afterward, MX (large granules dispensing certain nutrients), SJJXWS (a water-retaining agent), and AF (a nutrient conditioner) additives were added to study the physical, chemical, and agronomic properties of functional soil, such as the emergence rate and weight of plants. The results showed the high pH and conductivity of functional soil, implying alkaline soils with high salinity. The contents of organic matter and available phosphorus and potassium were relatively high, indicating its high nutrient content. Further analysis revealed that the MX was the key factor affecting functional soil’s electrical conductivity and evaporation, and thus, the corresponding plant emergence rate, plant weight, and other related indicators. The influence of each factor on the corresponding plant emergence rate, plant weight, and other indicators of functional soil was arranged in the order of MX (large granules dispensing certain nutrients), SJJXWS (a water-retaining agent), and AF (a nutrient conditioner). The optimum additive ratio in functional soil was 0.45 t·hm^-2^ of MX, 0.12 t·hm^-2^ of SJJXWS, and 1.65 t·hm^-2^ of AF. The results of this study provide a theoretical basis for further development of functional soil for ecological cycle restoration purposes.

## 1 Introduction

Coal combustion accounts for more than one-third of global power generation (Usman et al., 2022), leading to the annual production of about 800 million tons of fly ash, and China is ranked as the country with the largest fly ash production in the world (Wang et al., 2016). By coal burning, roughly 80% of coal ash leaves the furnace carried out by the flue gas, and the name ‘fly’ ash is inspired by this process (Ghodeswar and Oliver, 2022). Besides its enormous amount, the management and disposal of fly ash are inappropriate, leading to further deterioration of water and soil environments. Moreover, the excessive mining of stone materials and coal resources depletes resources, so mine environments urgently need structural restoration (Li and Lin, 2022; Zhang et al., 2022). Therefore, large-scale application and utilization of industrial solid waste fly ash (Mathapati et al., 2022) are of particular importance and may solve environmental problems and help restore sensitive localities.

Many studies support the utilization of fly ash on the global level, including its applications in building materials and agricultural production (Chen et al., 2022; Ou et al., 2022; Rid et al., 2022; Çelik et al., 2022). In agriculture, fly ash is mainly used as a soil amendment to improve physical properties (Islam and Islam, 2022). As an amendment, it can increase crop yield (Varshney et al., 2022), while as a fertilizer, it can help leguminous species to fix atmospheric nitrogen (Kumar and Kumar, 2022). A comparative field experiment on sunflower planting with fly ash, SAP, and PAM as modifiers showed that they effectively improved the net photosynthetic rate, stomatal conductance, intercellular CO_2_ mole fraction, and the transpiration rate of plants (Xu et al., 2017). A pot experiment of planting Lespedeza with fly ash, desulfurized gypsum, and citric acid residue as modifiers in a certain proportion demonstrated that the plant growth, leaf photosynthetic characteristics, and chlorophyll mass fraction increased at varying degrees (Yin et al., 2020). Fly ash, dolomite, gypsum, and potassium sulfate were mixed in a certain proportion for high-temperature roasting and then mixed with 2% CaCl_2_ to make a soil conditioner for planting sweet potatoes in the field test. The pH of modified soil moderately increased, and the sweet potato yield, plant weight, and nutritional components were also improved (Huang et al., 2021). However, these examples (only a very small amount of fly ash was used as an improved additive) represent a small-scale utilization of fly ash, which cannot quickly solve the problem of large fly ash accumulation. The basic components of an ordinary tillage soil layer (the main types of tillage are deep tillage, no-till, reduced tillage, and others.) are SiO_2_ and Al_2_O_3_ (Saaltink et al., 2014), similar to the composition of the raw fly ash used in the experiment (**Table 1**). The heavy metal content of the raw material (**Table 2**) meets the control index requirements of ecological restoration risk elements (**Table 3**) (Ministry of Civil Affairs of the People's Republic of China, 2021), and it is feasible to use fly ash as the main substrate for functional soil in ecological restoration. Therefore, using fly ash as the main matrix to explore the physical and chemical properties of functional soil and planting effects may be important for large-scale utilization of fly ash and ecological restoration.

**Table 1:** Composition analysis of raw materials (XRF unit: %).

Name	SiO_2_	Al_2_O_3_	CaO	Fe_2_O_3_	K_2_O	MgO	Na_2_O	Loss
Fly ash	44.8	22.6	6.2	5.7	1.7	1.8	1.5	15.7

**Table 2:** Heavy-metal content and index grade of raw materials.

Name	Heavy-metal content of fly ash (mg·kg^-1^)/ Control index level
Mercury (Hg)	0.572/II
Arsenic (As)	7.380/I
Lead (Pb)	56.000/I
Cadmium (Cd)	0.570/II
Chromium (Cr)	32.000/I
Copper (Cu)	42.000/I
Zinc (Zn)	47.000/I
Nickel (Ni)	22.000/I

**Table 3:** Requirements for the risk element content control indexes of fly ash for soil ecological restoration.

Project	Requirements for risk element content control indicators		
	I	II	III
Mercury (Hg, By element) , mg·kg^-1^	γ<0.5	0.5≤γ<1.0	1.0≤γ<2.0
Arsenic (As, By element) , mg·kg^-1^	γ<20	20≤γ<40	40≤γ<100
Lead (Pb, By element) , mg·kg^-1^	γ<70	70≤γ<170	170≤γ<400
Cadmium (Cd, By element) , mg·kg^-1^	γ<0.3	0.3≤γ<0.6	0.6≤γ<1.5
Chromium (Cr, By element) , mg·kg^-1^	γ<150	150≤γ<250	250≤γ<800
Copper (Cu, By element) , mg·kg^-1^	γ<50	50≤γ<100	100≤γ<200
Zinc (Zn, By element) , mg·kg^-1^	γ<200	200≤γ<250	250≤γ<300
Nickel (Ni, By element) , mg·kg^-1^	γ<60	60≤γ<100	100≤γ<190

**Figure 1:**
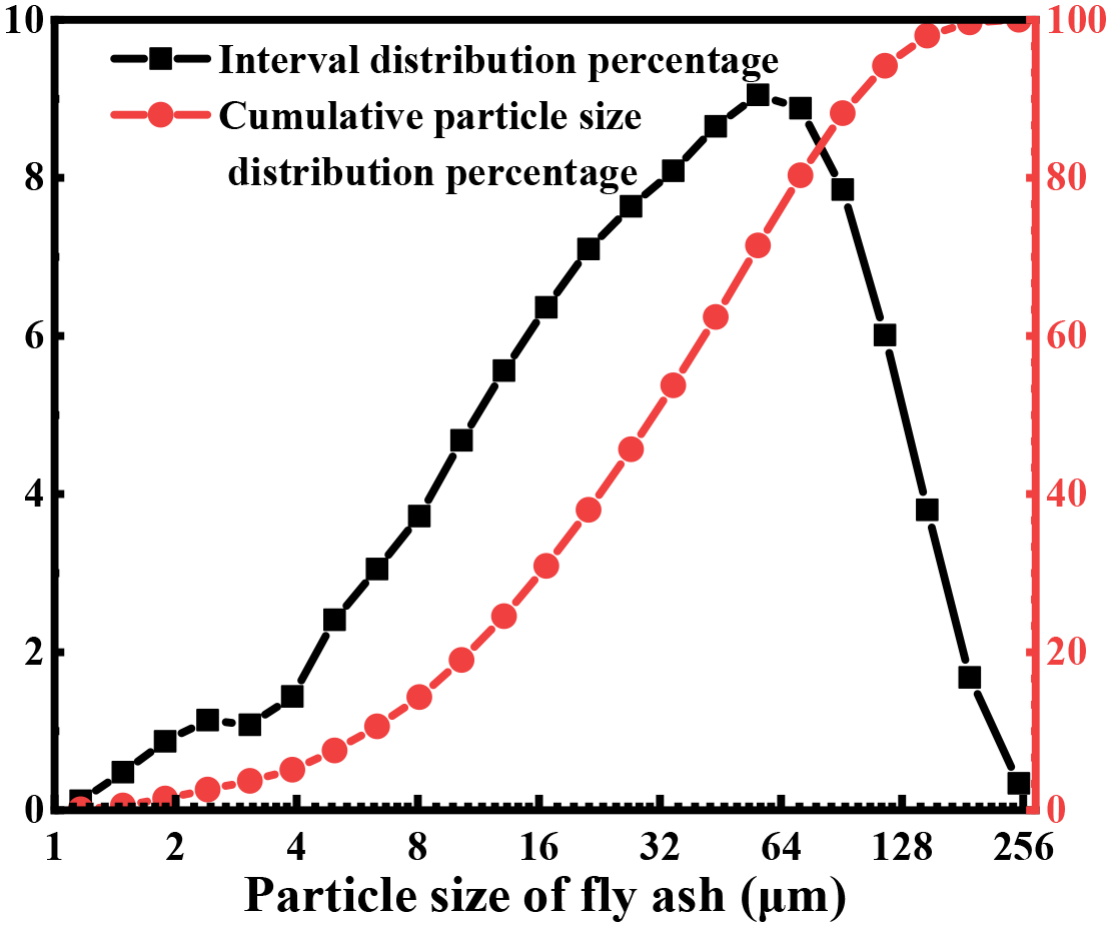
Particle size distribution of fly ash.

According to the limitation of fly ash as a functional soil, the addition of MX (large granules dispensing certain nutrients), SJJXWS (a water-retaining agent), and AF (a nutrient conditioner) additives may change the physical properties and composition of fly ash to enhance the effect of functional soil further. As the particle size of fly ash is small (**Figure 1**), MX was applied to modify the void structure of fly ash to improve the respiration of crop roots in functional soil; SJJXWS can improve the water retention capacity of functional soil, while AF can increase the nutrient content of functional soil and promote plant growth. pH, conductivity, the contents of organic matter, available phosphorus and potassium, evaporation capacity, seedling emergence rate, plant height, and plant weight of the substrate were studied, and the key factors affecting the water holding capacity, nutrient content, and plant agronomic properties of the substrate were discussed to determine the optimal proportion of the substrate and provide a certain theoretical basis for the ecological restoration of fly ash.

## 2 Materials and methods

### 2.1 Raw materials

Based on orthogonal and pot experiments, MX (large granules dispensing certain nutrients), SJJXWS (a water-retaining agent), and AF (a nutrient conditioner) were added to fly ash in different proportions to prepare functional soil for ecological restoration. The fly ash used in this experiment was obtained from an enterprise in Ningxia, while MX, SJJXWS, and AF were all self-prepared. MX consists of large granules releasing certain nutrients, containing a certain amount of cellulose; SJJXWS is a water-retaining agent, and AF is a nutrient conditioner that provides nitrogen. The basic physical and chemical properties of fly ash are shown in **Table 4**.

**Table 4:** Basic physicochemical parameters of raw materials.

Raw material	Moisture content %	pH	EC/(µs·cm^-1^)	Organic matter content/(g·kg^-1^)	Available phosphorus content (mg·kg^-1^)	Available potassium content (mg·kg^-1^)
Fly ash	1.13	9.18	4870.00	63.40	13.85	132.33

### 2.2 Orthogonal experimental design

In this experiment, we used the orthogonal experimental design. This method uses a constructed orthogonal matrix to analyze experimental data and can clarify the weights of each parameter on the result according to the experimental results to find better parameter matching; the biggest advantage is that the optimization of the required experimental data is straightforward, effectively shortening the time required for optimization. Considering the characteristics of raw materials, we designed three factors (MX, SJJXWS, AF) and five levels of orthogonal experiments, with four parallel replications at each factor level. The factor level table is shown in **Table 5**, and the content level of additives related to functional soil is shown in **Table 6**.

**Table 5:** Factor level table.

Level	Factor A (MX)	Factor B (SJJXWS)	Factor C (AF)
1	0.45	0.03	0.45
2	0.75	0.06	0.75
3	1.05	0.09	1.05
4	1.35	0.12	1.35
5	1.65	0.15	1.65

**Table 6:** Composition of functional soil additives by an orthogonal test.

Number	A	B	C		Number	A	B	C
1	1	1	1		14	3	4	1
2	1	2	2		15	3	5	2
3	1	3	3		16	4	1	4
4	1	4	4		17	4	2	5
5	1	5	5		18	4	3	1
6	2	1	2		19	4	4	2
7	2	2	3		20	4	5	3
8	2	3	4		21	5	1	5
9	2	4	5		22	5	2	1
10	2	5	1		23	5	3	2
11	3	1	3		24	5	4	3
12	3	2	4		25	5	5	4
13	3	3	5					

### 2.3 Pot experiment

Oat grass (*Avena fatua L.*) (Gang and Zhang, 2019), a gramineous plant species that exhibits salt-alkali resistance, cold resistance, and drought resistance, was selected as the test grass species, exhibiting rapid growth and quickly restoring green on the mountain slope. The selected main matrix was fly ash after the removal of heavy metals. The content of the components is shown in **Table 1**. The content of heavy metals (**Table 2**) meets the risk element control indicators required for ecological restoration (**Table 3**) (Ministry of Civil Affairs of the People's Republic of China, 2021). The content of heavy metals is determined according to the specification (Ministry of ecological environment of the people's Republic of China, 1998; General Administration of quality supervision, inspection and Quarantine of the people's Republic of China and China National Standardization Administration Committee, 2008a; General Administration of quality supervision, inspection and Quarantine of the people's Republic of China and China National Standardization Administration Committee, 2008b; Ministry of ecological environment of the people's Republic of China, 2019). The total required mass of fly ash is calculated based on the area (0.067 hm^2^), thickness (40 cm), and density of fly ash. Then, it was proportionally reduced to 1 kg, different additives (MX, SJJXWS, and AF) were added according to the horizontal factor table, and the corresponding pot experiment was performed to find the additive with the optimal effect. Uniformly mixed functional soil was put into a flower pot (the size of the pot was 16 cm in the upper diameter, 12 cm in the lower diameter, and 11.5 cm in height.). In each test flower pot, 30 plant seeds were placed 1 - 2 cm deep into the soil and regularly watered weekly to maintain a water content above 15%. The emergence rate represents the percentage of the number of seedlings in each pot after 30 days of planting. Photographs of the actual situation for records were taken; then, the relevant performance was measured, and the data were summarized and analyzed for outcomes. Each data group represents the average standard deviation value of four parallel experiments.

### 2.4 Determination method

The moisture content was determined by drying in an oven at a constant temperature (Ministry of Agriculture of the People's Republic of China, 2016); At room temperature of 25 °C and an initial moisture content of 30%, the water evaporation amount was measured by weighing the experimental sample with an electronic balance at a fixed time. The pH of the experimental sample was determined potentiometrically by reading the pH value according to the potential difference measured by a pH meter (Ministry of Agriculture of the People's Republic of China, 2006); A conductivity meter was used to measure the resistance and the conductivity of the experimental samples (Ministry of environmental protection of the people's Republic of China, 2016). The organic matter content of the experimental sample was determined by the burning loss method using an analytical balance to determine the weight loss upon ignition (Environmental protection of the people's Republic of China, 2015). The contents of available phosphorus and potassium in the experimental samples were determined using combined extraction colorimetry with an ultraviolet spectrophotometer (Ministry of Agriculture of the People's Republic of China, 2010). The emergence rate was determined using the counting method. The plant height and root length were measured with the ruler method. Chlorophyll was directly measured by a chlorophyll meter (Xu et al., 2021). The plant weight was measured using an analytical balance. The content of heavy metals in fly ash is determined by the standard soil-related heavy metal detection method. For Hg, atomic fluorescence spectrometry was adopted (General Administration of quality supervision, inspection and Quarantine of the people's Republic of China and China National Standardization Administration Committee, 2008a), with a detection limit of 0.002 mg·kg^-1^, for the sake of accuracy, the relative deviation should not exceed 12%, and the absolute value of relative error should not exceed 5%. For As, we adopted atomic fluorescence spectrometry (General Administration of quality supervision, inspection and Quarantine of the people's Republic of China and China National Standardization Administration Committee, 2008b), with a detection limit of 0.001 mg·kg^-1^. The relative deviation was below 7%, and the absolute value of the relative error did not exceed 5%. For Pb, we used atomic absorption spectrometry (Ministry of ecological environment of the people's Republic of China, 2019), with a detection limit of 10 mg·kg^-1^; the relative deviation was not more than 5.4%, and the absolute value of the relative error was not more than 3%; Cd was determined using atomic absorption spectrometry (Ministry of ecological environment of the people's Republic of China, 1998), with a detection limit of 0.01 mg·kg^-1^, the relative deviation did not exceed 3.6%, the absolute value of the relative error was below 3.6%; Cr was measured using atomic absorption spectrometry (Ministry of ecological environment of the people's Republic of China, 2019), with a detection limit of 4 mg·kg^-1^, the relative deviation was not more than 6.8%, and the absolute value of the relative error was below 2.6%. Atomic absorption spectrometry (Ministry of ecological environment of the people's Republic of China, 2019) was used to determine the concentration of Cu, with a detection limit of 1 mg·kg^-1^, the relative deviation was not more than 4.0%, and the absolute value of the relative error was below 2.7%; Zn was determined using atomic absorption spectrometry (Ministry of ecological environment of the people's Republic of China, 2019), with a detection limit of 1 mg·kg^-1^, the relative deviation did not exceed 4.7%, and the absolute value of the relative error does not exceed 1.3%. Ni was determined using atomic absorption spectrometry (Ministry of ecological environment of the people's Republic of China, 2019), with a detection limit of 3 mg·kg^-1^, the relative deviation was not more than 4.2%, and the absolute value of the relative error was less than 4.1%.

The fly ash composition was determined using X-ray fluorescence (XRF). Specifically, the sample was prepared by a powder compression method, measured directly using a wavelength-dispersive X-ray fluorescence spectrometer, and the measured components were calculated according to the fluorescence intensity of the element (Ministry of Land and Resources of the People's Republic of China, 2016). The data were analyzed and processed using Origin 2018 and SPSS 26.0 statistical software. The experimental equipment and model numbers are shown in **Table 7**.

**Table 7:** Experimental equipment and device models.

Equipment	Model
Precision pH meter	P901L
Electric blast drying box	101-3AB
Muffle furnace	KSL-1400X
Chlorophyll analyzer	TYS-B
X-fluorescence spectroscopy analyzer	AxiosPW4400
Electronic balances	YP30002
Laser particle size analyzer	Bettersize2000B&E
Analyze the balance	MS104TS-2
Conductivity meter	DDSJ-308F
Dual-function water bath thermostat	JTD-6000
UV spectrophotometer	UV2700
Atomic absorption spectrometer	SavantAA
Atomic fluorescence spectrometer	SK-2003A

## 3 Results

The median particle diameter (D50) fly ash is only 39.45 µm, as determined using a particle size analyzer. The particle size distribution diagram is shown in **Figure 1**, indicating that the particle size of fly ash is almost three times lower than that of the ordinary soil tillage layer (110 µm) (Jayarathne et al., 2020).

### 3.1 Evaporation characteristics of functional soil

**Figure 2:**
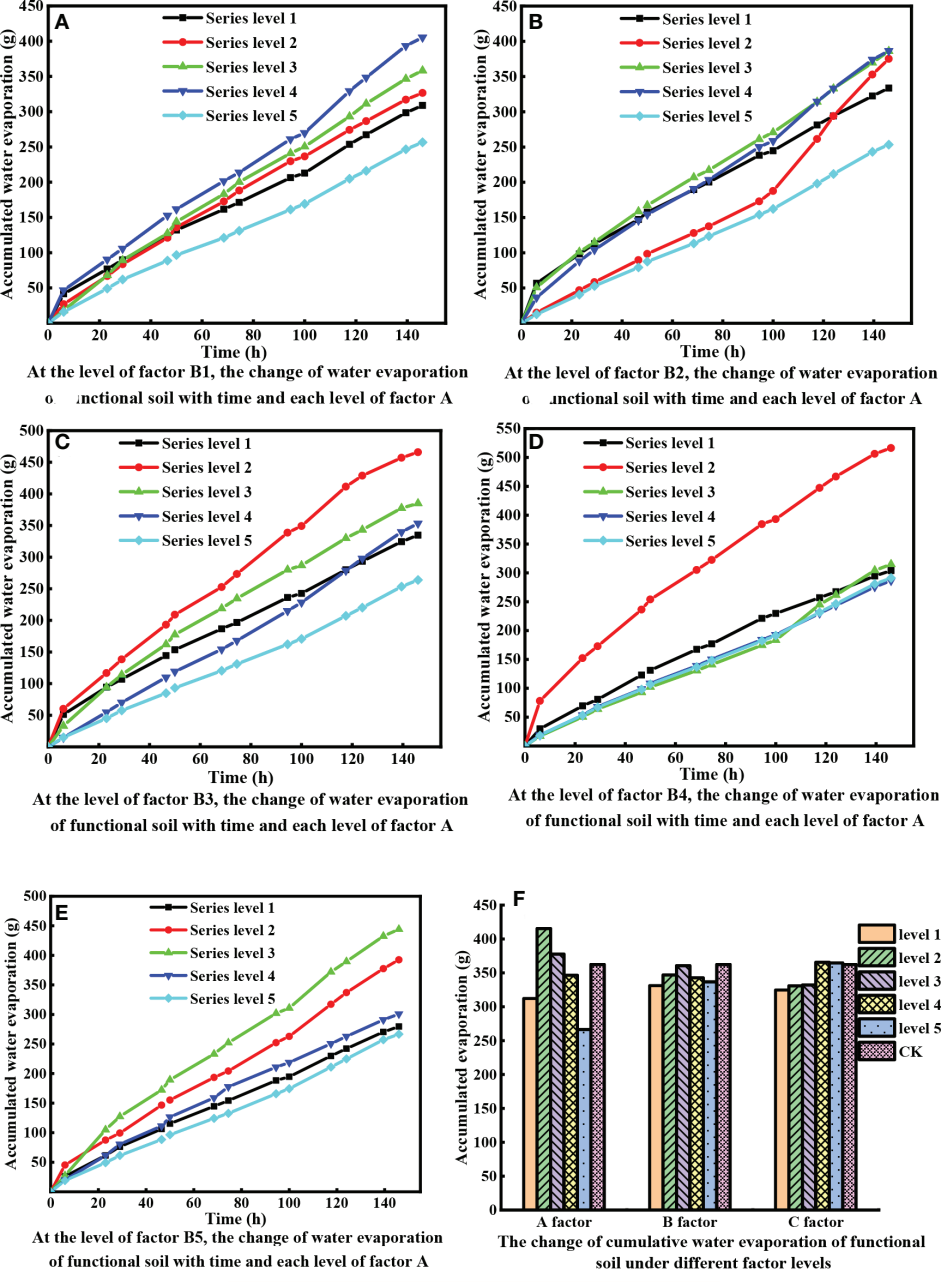
Evaporation characteristics of the functional soil.

Water capacity is an important physical property of soil, and strong water retention is suitable for plant growth in arid areas (Ghassemi-Golezani and Farhangi-Abriz, 2022). It can be determined through evaporation experiments based on the added amount of SJJXWS (0.03 ~ 0.15 t·hm^-2^). The evaporation experiment was divided into 5 groups, and each group was discussed according to the MX addition level. **Figures 2A–E** shows the effect of different MX ratios (0.45, 0.75, 1.05, 1.35, and 1.65 t·hm^-2^) on the evaporation characteristics of the functional soil when the SJJXWS amount is 0.03, 0.06, 0.09, 0.12, and 0.15 t·hm^-2^, respectively. The evaporation experiment of the functional soils also adopts the orthogonal experimental method, but the data analysis is carried out from the following perspectives: **Figure 2A** shows the evaporation of water from the functional soil with the change in the MX content (at 5 levels of factor A from small to large) at an SJJXWS concentration of 0.03 t·hm^-2^ (the first level of factor B); **Figure 2B** shows the water evaporation from the functional soil with the change of MX content (at 5 levels of the A factor from small to large) at an SJJXWS concentration at the second level of 0.06 t·hm^-2^ (the second level of the B factor); **Figure 2C** shows the evaporation of water from the functional soils with the change in the MX content (at 5 levels of factor A from small to large) at an SJJXWS concentration of 0.09 t·hm^-2^ (the third level of factor B); **Figure 2D, E**, and so on. **Figure 2F** shows the cumulative water evaporation from functional soils under the influence of the three factors (MX, SJJXWS, and AF) and their levels.

According to the evaporation experiment, the results show that the cumulative evaporation of all functional soils gradually increases with the evaporation time. According to **Figures 2A–E**, at the same amount of SJJXWS added to the functional soil matrix when the cumulative water evaporation of the functional soil is high, the MX content is 0.75 t·hm^-2^; when the cumulative water evaporation of functional soil is low, the MX content is 1.65 t·hm^-2^. This shows that the cumulative evaporation of functional soil water decreases with the MX content. **Figure 2F** shows that among various factors influencing the average evaporation, factor A induces the largest change, and the range is 266.33 - 415.33 g, indicating that MX has the greatest impact on the water evaporation characteristics of the functional soil. The average evaporation without the influence of various factors in the blank control group is much larger than for factor A (MX), level 5.

### 3.2 Physical and chemical characteristics of the functional soil

**Figure 3:**
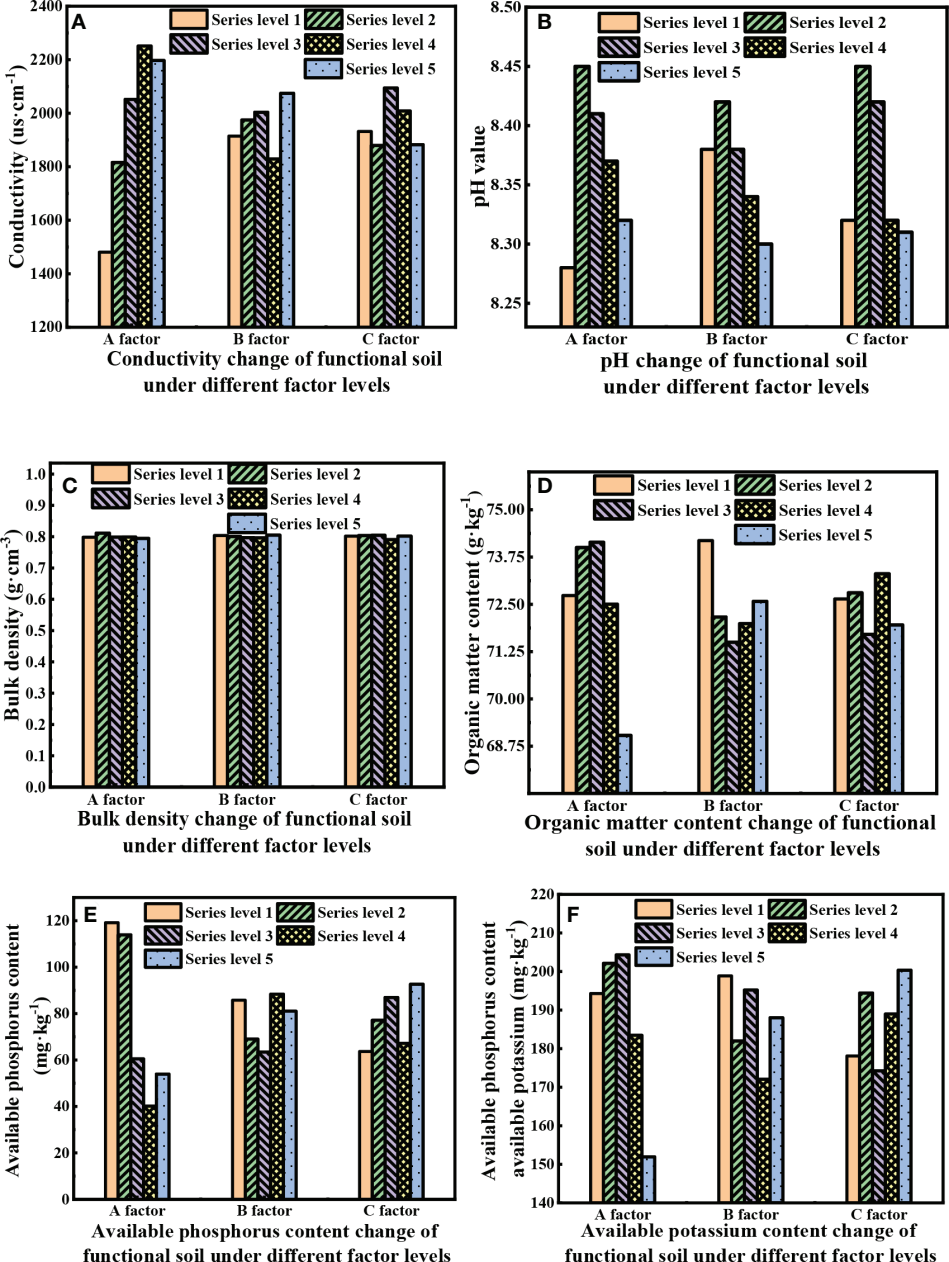
The physical and chemical characteristics of functional soils vary with the influence of different factors.

Under the influence of horizontal factors, the chemical characteristics of the functional soil change to varying degrees, as shown in **Figure 3**.

**Figure 3A** shows that different factors affect the matrix conductivity differently. The conductivity of the functional soil in the blank control group without horizontal factor interference is 1846 µs·cm^-1^. The functional soil conductivity variation range under the horizontal influence of factors (factor A, factor B, and factor C) is 1481 - 2251, 1829 – 2075, and 1879 - 2094 µs·cm^-1^, respectively. However, factor A (MX) has a greater impact on matrix conductivity, with an extreme value of 770.4, while the extreme values of B (including SJJXWS) and C (AF) are 245.15 and 214.95, respectively. The impact of factor A is about 3.14 - 3.58 times higher than that of factors B and C. With the quantitative increase in factor A, the conductivity of the substrate first increases and then decreases. When the content of the A factor is 4 levels (1.35 t·hm^-2^), the conductivity reaches the highest value of 2250.95 µs·cm^-1^. The overall trend of factor B and factor C is not obvious, and the average conductivity value is 1959.25 µS·cm^-1^. The maximum substrate conductivity induced by factor B and factor C is 2074.45 µS·cm^-1^ at level 5 (0.15 t·hm^-2^) and 2094.35 µS·cm^-1^ at level 3 (1.05 t·hm^-2^), respectively, but it is less than the conductivity value of factor A at level 4 of 2250.95 µs·cm^-1^.

**Figure 3B** shows that the pH of the fly ash control is 8.59, and the pH change range according to the level of each factor (factor A, factor B, and factor C) is 8.28 - 8.45, 8.30 - 8.42, and 8.31 - 8.45, respectively. Among them, the average pH value of factor A (MX), factor B (SJJXWS), and factor C (AF) is 8.36; the extreme value of factor A is 0.17, the extreme value of factor B is 0.12, and the extreme value of factor C is 0.14. The extreme value is the largest for factor A, followed by factors C and B. The influence of the three factors on the pH value of the substrate first increases and then decreases with the content level, and the pH value reaches the highest value at level 2. The overall pH value fluctuates between 8.28 - 8.45, with a small range. At level 1 of factor A, level 5 of factor B, and level 5 of factor C, the pH value is close to the neutral level within their respective factor levels. Under the influence of the level of factor A, the pH of the functional soil exhibits a large variation range and follows a certain law. The pH value first increases and then decreases with the level of factor A. Other factor levels exhibit little influence on the pH variation range.

**Figure 3C** shows that the change of unit weight under each factor level is 0.794 - 0.811, 0.796 - 0.805, and 0.790 - 0.805 g·cm^-3^, respectively. The overall variation range is small, and the unit weight decreases and then increases with the level of factor B. The reason is that SJJXWS is a water-retention agent that can change the compactness of functional soils to a certain extent.

**Figure 3D** shows that the content of organic matter in the fly ash control is 72.07 g·kg^-1^, and the change in the content of organic matter under each factor level is 69.03 - 74.14, 71.50 - 74.19, and 71.71 - 73.31 g·kg^-1^. The variation range of the content of organic matter under the different levels of factor A and factor B, is large and changes regularly. The organic matter content increases first and then decreases with the levels of factor A and factor B.

**Figure 3E** shows that the effective phosphorus content in the fly ash control is 7.53 mg·kg^-1^, and the variation range of the effective phosphorus content with the level of factors A, B, and C is 40.11 - 119.14, 63.42 - 88.38, and 63.67 - 92.65, respectively, of which the variation range of factor A is the largest and changes regularly, that is, the effective phosphorus content in the matrix first decreases and then increases with the level of factor A.

**Figure 3F** shows that the available potassium in the fly ash control is 138.53 mg·kg^-1^, and the variation range of the available potassium content with the level of factors A, B, and C is 151.94 - 204.31, 172.06 - 198.87, and 174.26 - 200.35, respectively, of which the change range of factor A is the largest and regular, that is, the content of available potassium in the matrix first increases and then decreases with the level of factor A.

### 3.3 Relevant agronomic characters of plants corresponding to the functional soil

**Figure 4:**
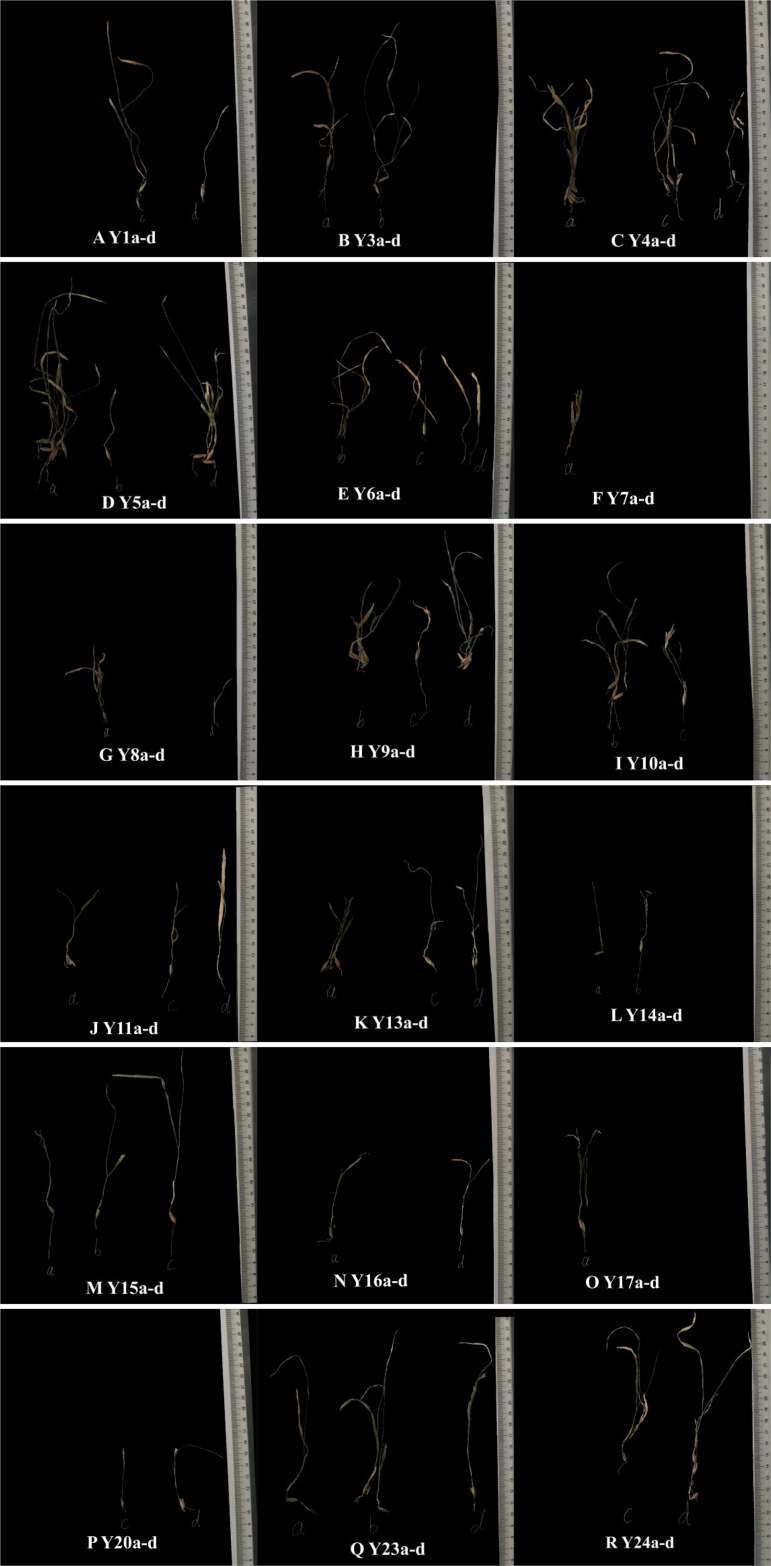
Final growth status of test plants after 30 days.

**Figure 5:**
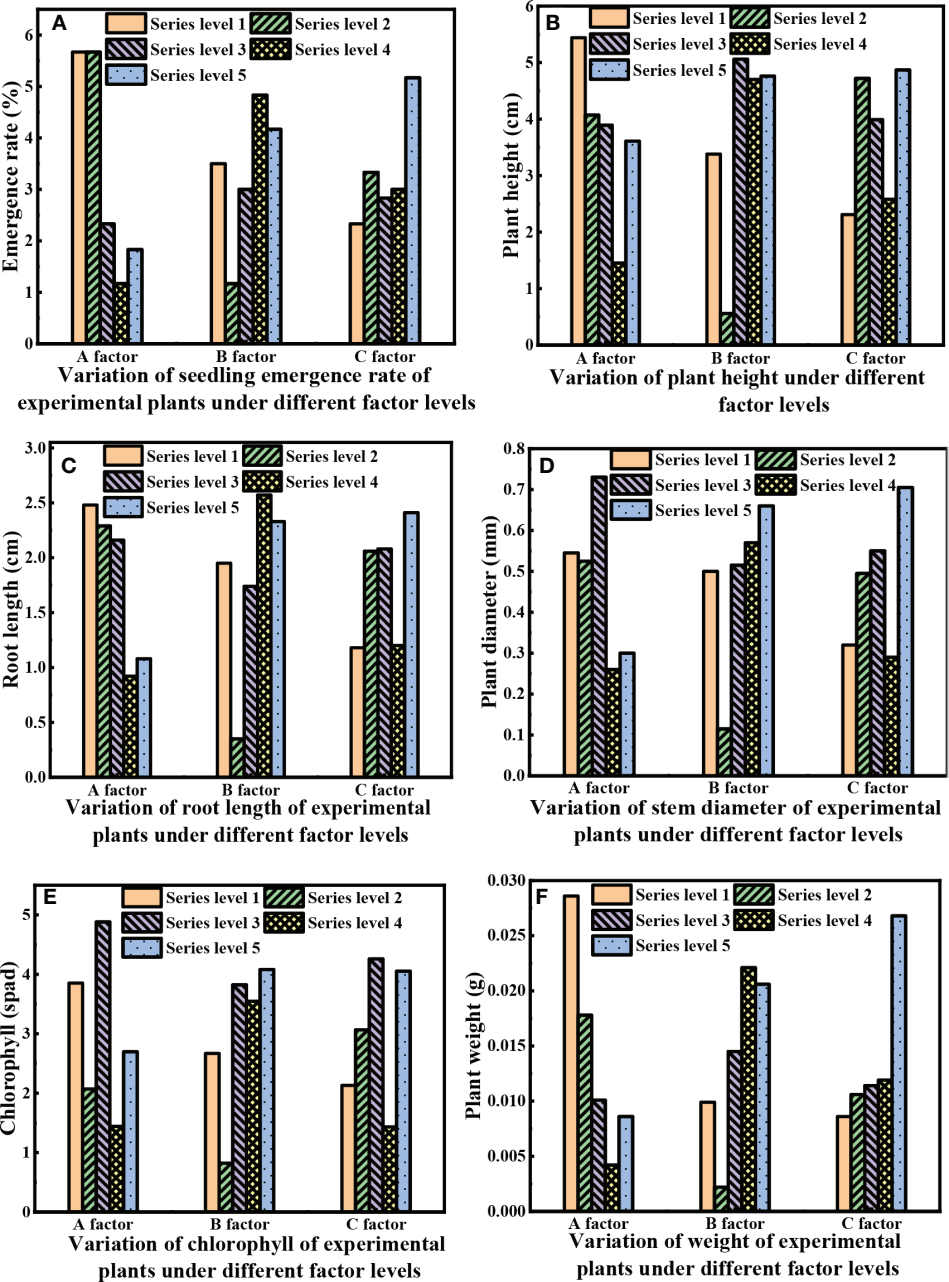
Variation of agronomic characters of test plants with factor level.

The final growth status of the plant on the 30^th^ day of sowing is shown in **Figure 4**. We determined the seedling emergence rate, stem length, root length, diameter, chlorophyll, and plant weight. The measurement method is shown in Section 2.4. **Figures 5A–F** shows the change charts of the plant emergence rate, stem length, root length, stem diameter, chlorophyll, and plant weight due to different factor levels. Among the levels of various matrix factors, the indexes of various agronomic traits of plants are quite different, and the change in the regularity of factor A is more obvious than that of the other two factors. **Figures 5A–C, F** shows that the seedling emergence rate, stem length, root length, and the plant weight corresponding to the substrate first decrease and then increase at the level of factor A. Under the influence of the MX factor, the variation ranges of the seedling emergence rate, stem length, root length, and plant weight are 1.17 - 5.67%, 1.45 - 5.44 cm, 0.92 - 2.48 cm, and 0.0042 - 0.0286 g, respectively; when the MX content is 0.45 t·hm^-2^, it reaches the highest value of 5.67%, 5.44 cm, 2.48 cm, and 0.0286 g, respectively. It shows a dose-dependent relationship between the MX amount and the plant emergence rate, stem length, root length, and plant weight. The other two factors (SJJXWS and AF) do not have significant, regular changes in the stem length and root length of plants, and SJJXWS does not exhibit significant regularity changes in plant emergence rates. However, with the increase in the AF concentration, the emergence rate and plant weight of plants gradually increase, and the variation range is 2.33-5.17% and 0.0086-0.0268g, respectively, indicating that AF has a certain effect on plant growth. **Figure 5D** shows that SJJXWS has a greater impact on the plant stem thickness, and it shows an overall growth trend. The other two factors (MX and AF) do not have significant regular changes in the stem thickness of the plant. It can be seen from **Figure 5E** that the regularity of chlorophyll is not significant.

The emergence rate, stem length, diameter, chlorophyll, and plant weight show that the effect of factor A (MX) is stronger than that of the other two factors (factor B for SJJXWS and factor C for AF). From the perspective of plant weight, the higher the content of factor A, the lower the plant weight. When the content of factor A is at level 1, it is most conducive to plant growth; for factor C, the higher the content, the higher the plant weight. That is, when the content of factor C is at level 5 within the experimental range, it is most conducive to plant growth.

## 4 Discussion

MX is the main factor affecting the evaporation of functional soil water because MX contains cellulose, and cellulose contains hydrophilic hydroxyl groups, exhibiting a strong flocculation effect and water retention ability, retaining the water in the functional soil and limiting evaporation.

Soil conductivity is one of the basic physical properties of soil, and to a certain extent, it represents the abundance and shortage of salt ions, such as Ca^2+^, Mg^2+^, and K^+^, in soil and is one of the important indicators of soil fertility (Yin et al., 2014). According to the previous research (Guan et al., 2021), the conductivity of normal soil is within the range of 500 – 3200 µs·cm^-1^, so the conductivity of the test substrate is in line with the conductivity range of normal soil. pH, like conductivity, belongs to the basic chemical properties of soil, and it represents the abundance of salt ions in soil and is also one of the important indicators of soil fertility (Li et al., 2020). According to the reported research (Chao and Juan, 2021), the soil pH value of normal and undeveloped grassland is 7.84, and the pH value range of this test is 8.28 - 8.45, which is obviously higher. However, compared with the blank control group without the influence of factor level, the pH of functional soil is relatively low. Factor A is the main factor affecting the pH of functional soil because MX, as a particle conditioner, can improve soil agglomeration, thus affecting the particle size of soil, effectively improving the soil pH to a certain extent. Moreover, MX and AF, as nutrient modulators, can change the pH of functional soils by undergoing certain nitrification reactions (Cristian Kofi et al., 2020).

The content of organic matter content in soil is one of the important indicators of soil fertility (Zhang et al., 2020). Factors A and B can affect the organic matter content of functional soils because MX, as a particle conditioner, also contains certain organic matter, changing the organic matter content of the matrix; SJJXWS is a water retention agent that can change the distribution of good/anaerobic organisms in functional soils by changing the moisture distribution, thereby changing the rate of mineralization and differentiation of organic matter in soils (Liao et al., 2012), affecting the organic matter content of functional soils.

Available phosphorus and potassium in the soil are indispensable and important nutrients in the growth process of plants (Peng et al., 2022). The reason why factor A has a significant impact on the available phosphorus and potassium contents in functional soils is that MX is used as a granulate adjustment, which can have a certain impact on soil agglomeration; furthermore, soil agglomeration can maintain the nutrient composition of the functional soil, so that the stronger the agglomeration effect, the higher the nutrient content and the higher the fertility of the functional soil (Zhao et al., 2021). Thus, the content of available phosphorus and available potassium in the functional soil exhibits a certain change.

The reason why factor A has become an important factor affecting the emergence rate, stem length, root length, plant weight, etc., of plants is that a small amount of MX does not only increase the amount of certain nutrients in the matrix but also acts as a granule adjustment that changes the matrix void to reduce the functional soil compaction phenomenon; however, an excessive application of MX may increase the conductivity of the matrix, thereby inhibiting the growth of oat grass. Factor B has become an important factor affecting the thickness of the plant stem because SJJXWS, as a water retention agent, can increase the water-retention capacity of the functional soil to a certain extent. That fosters sufficient water absorption and leads to the thick stem, and the decreasing phenomenon for the first two levels may be due to the low content of the first two levels, which has little impact on the actual plant stem diameter, while the larger stem diameter of the later plants originates from the higher plant water content due to the higher elemental level. Factor C is an important factor affecting the plant emergence rate and plant weight because AF, as a nutrient, can provide plants with certain nutrients to promote plant growth.

The conductivity, evaporation, emergence rate, stem length, root length, and plant weight of the functional soil were analyzed to explore the effects of various factors on conductivity, evaporation, emergence rate, stem length, root length, and plant weight. The results of the experimental range analysis are shown in **Table 8**.

**Table 8:** Experimental range analysis results.

Calculation items		K1	K2	K3	K4	K5	R
Conductivity	A	1480.55	1816.00	2051.75	2250.95	2197.00	770.40
	B	1914.50	1974.85	2003.60	1828.85	2074.45	245.60
	C	1931.55	1879.40	2094.35	2008.40	1882.55	214.95
Evaporation capacity	A	312.19	415.33	377.69	346.45	266.33	149.00
	B	331.20	346.93	360.57	342.56	336.72	29.37
	C	324.60	330.94	332.04	365.63	364.78	41.03
Emergence rate	A	5.67	5.67	2.33	1.17	1.83	4.50
	B	3.50	1.17	3.00	4.83	4.17	3.67
	C	2.33	3.33	2.83	3.00	5.17	2.83
Stem length	A	5.44	4.07	3.89	1.45	3.61	4.00
	B	3.38	0.56	5.06	4.70	4.76	4.51
	C	2.31	4.72	3.99	2.58	4.87	2.56
Root length	A	2.48	2.29	2.16	0.92	1.08	1.56
	B	1.95	0.35	1.74	2.57	2.33	2.22
	C	1.18	2.06	2.08	1.20	2.41	1.24
Plant weight	A	0.0286	0.0178	0.0101	0.0042	0.0086	0.0243
	B	0.0099	0.0022	0.0145	0.0221	0.0206	0.0198
	C	0.0086	0.0106	0.0114	0.0119	0.0268	0.0182

The analysis results in this work show that the influence of each factor on each index of the functional soil is different. The influence on the conductivity follows the order of MX> SJJXWS > AF; the intensity influence on the evaporation characteristics of functional soil is MX > SJJXWS > AF; the emergence rate of the corresponding plants in the functional soil should be MX > SJJXWS > AF; the influence of each factor on the stem length from big to small is SJJXWS – MX - AF; the influence on the root length of corresponding plants in the functional soil is SJJXWS > MX > AF. The influence on the plant weight of the functional soil is arranged in the order of MX > SJJXWS > AF. The results of this study show that MX has the greatest impact on the conductivity, evaporation, corresponding plant emergence rate, and plant weight of the functional soil because it can improve the porosity of the functional soil to a certain extent, but the excessive application of MX may increase the conductivity of the matrix, thereby inhibiting the growth of oat grass and reducing its emergence rate and plant weight.

In this paper, the best proportion of functional soil is determined according to the comprehensive balance method. The comprehensive balance method is used to calculate and analyze the single index first to obtain the best combination of the factor levels and then comprehensively balance the importance of each index, the primary and secondary factors, and the advantages and disadvantages of the level, finally determining the overall best factor level combination. **Table 8** shows that the best average performance level of each factor, including conductivity, evaporation experiment, emergence rate, plant weight, stem length, and root length, is A_1_B_4_C_2_, A_5_B_1_C_1_, A_1_B_4_C_5_, A_1_B_4_C_5_, A_1_B_3_C_5_, and A_1_B_4_C_5,_ respectively. By comprehensively considering the characteristics of electrical conductivity, evaporation, emergence rate, plant weight, stem length, and root length of the functional soil, it is concluded that the best ratio of functional soil in this study is A_1_B_4_C_5_ - MX (0.45 t ·hm^-2^), SJJXWS (0.12 t ·hm^-2^) AF (1.65 t ·hm^-2^).

## 5 Conclusion

Compared with the original fly ash, the pH value of functional soil is relatively low, but it still belongs to alkaline functional soils. Relatively low electrical conductivity indicates that the salt content in the functional soil is low, while the contents of organic matter, available phosphorus, and available potassium are relatively high, indicating a high nutrient content of the functional soil and providing nutrients for plants and promoting plant growth. Through the characterization of electrical conductivity, evaporation, pH, organic matter, available phosphorus, available potassium, seedling emergence rate, and plant weight, it can be seen that MX is the key factor affecting the characteristics of the functional soil. After analyzing the emergence rate of the corresponding plants in the functional soil, it was found that the order of influence of each factor was MX > SJJXWS > AF. According to the comprehensive analysis, the best proportion of the matrix functional soil is MX (0.45 t ·hm^-2^), SJJXWS (0.12 t ·hm^-2^), and AF (1.65 t ·hm^-2^). This study provides a relevant basis for the further development of ecological cycling functional soils.

## Data availability statement

The original contributions presented in the study are included in the article/supplementary material. Further inquiries can be directed to the corresponding author.

## Author contributions

Credit Author Statement TL: Writing-Original Draft, Investigation, Formal analysis, Visualization; FH: Writing-Review & Editing, Supervision, Project administration, Funding acquisition; XZ: Investigation, Formal analysis; JW: Investigation, Formal analysis; XD: Writing-Review; CA: Investigation, Supervision. All authors contributed to the article and approved the submitted version.

## Funding

Ningxia Hui Autonomous Region Key R&D Program Project (Grant No. 2021BEE02019).

## Conflict of interest

The authors declare that the research was conducted in the absence of any commercial or financial relationships that could be construed as a potential conflict of interest.

## Publisher’s note

All claims expressed in this article are solely those of the authors and do not necessarily represent those of their affiliated organizations, or those of the publisher, the editors and the reviewers. Any product that may be evaluated in this article, or claim that may be made by its manufacturer, is not guaranteed or endorsed by the publisher.
